# Seasonal influences on surface ozone variability in continental South Africa and implications for air quality

**DOI:** 10.5194/acp-18-15491-2018

**Published:** 2018-10-29

**Authors:** Tracey Leah Laban, Pieter Gideon van Zyl, Johan Paul Beukes, Ville Vakkari, Kerneels Jaars, Nadine Borduas-Dedekind, Miroslav Josipovic, Anne Mee Thompson, Markku Kulmala, Lauri Laakso

**Affiliations:** 1 Unit for Environmental Sciences and Management, North-West University, Potchefstroom, South Africa; 2 Finnish Meteorological Institute, Helsinki, Finland; 3 Department of Environmental Systems Science, ETH Zürich, Zürich, Switzerland; 4 NASA/Goddard Space Flight Center, Greenbelt, Maryland, USA; 5 Department of Physics, University of Helsinki, Helsinki, Finland

## Abstract

Although elevated surface ozone (O_3_) concentrations are observed in many areas within southern Africa, few studies have investigated the regional atmospheric chemistry and dominant atmospheric processes driving surface O_3_ formation in this region. Therefore, an assessment of comprehensive continuous surface O_3_ measurements performed at four sites in continental South Africa was conducted. The regional O_3_ problem was evident, with O_3_ concentrations regularly exceeding the South African air quality standard limit, while O_3_ levels were higher compared to other background sites in the Southern Hemisphere. The temporal O_3_ patterns observed at the four sites resembled typical trends for O_3_ in continental South Africa, with O_3_ concentrations peaking in late winter and early spring. Increased O_3_ concentrations in winter were indicative of increased emissions of O_3_ precursors from household combustion and other low-level sources, while a spring maximum observed at all the sites was attributed to increased regional biomass burning. Source area maps of O_3_ and CO indicated significantly higher O_3_ and CO concentrations associated with air masses passing over a region with increased seasonal open biomass burning, which indicated CO associated with open biomass burning as a major source of O_3_ in continental South Africa. A strong correlation between O_3_ on CO was observed, while O_3_ levels remained relatively constant or decreased with increasing NO_*x*_, which supports a VOC-limited regime. The instantaneous production rate of O_3_ calculated at Welgegund indicated that ~ 40 % of O_3_ production occurred in the VOC-limited regime. The relationship between O_3_ and precursor species suggests that continental South Africa can be considered VOC limited, which can be attributed to high anthropogenic emissions of NO_*x*_ in the interior of South Africa. The study indicated that the most effective emission control strategy to reduce O_3_ levels in continental South Africa should be CO and VOC reduction, mainly associated with household combustion and regional open biomass burning.

## 1 Introduction

High surface O_3_ concentrations are a serious environmental concern due to their detrimental impacts on human health, crops and vegetation (NRC, 1991). Photochemical smog, comprising O_3_ as a constituent together with other atmospheric oxidants, is a major air quality concern on urban and regional scales. Tropospheric O_3_ is also a greenhouse gas that directly contributes to global warming (IPCC, 2013).

Tropospheric O_3_ concentrations are regulated by three processes, i.e. chemical production–destruction, atmospheric transport, and losses to the surface through dry deposition (Monks et al., 2015). The photolysis of nitrogen dioxide (NO_2_) in the presence of sunlight is the only known way of producing O_3_ in the troposphere (Logan, 1985). O_3_ can recombine with nitric oxide (NO) to regenerate NO_2_, which will again undergo photolysis to regenerate O_3_ and NO. This continuous process is known as the NO_*x*_ -dependent photo-stationary state (PSS) and results in no net production or consumption of ozone (null cycle). However, net production of O_3_ in the troposphere occurs outside the PSS when peroxy radicals (HO_2_ and RO_2_) alter the PSS by oxidizing NO to produce “new” NO_2_ (Cazorla and Brune, 2010), resulting in net O_3_ production. The main source of these peroxy radicals in the atmosphere is the reaction of the hydroxyl radical (OH^•^) with volatile organic compounds (VOCs) or carbon monoxide (CO) (Cazorla and Brune, 2010).

O_3_ precursor species can be emitted from natural and anthropogenic sources. Fossil fuel combustion is considered to be the main source of NO_*x*_ in South Africa, which includes coal-fired power generation, petrochemical operations, transportation, and residential burning (Wells et al., 1996; Held et al., 1996). Satellite observations indicate a well-known NO_2_ hotspot over the South African Highveld (Lourens et al., 2012) attributed to industrial activity in the region. CO is produced from three major sources, i.e. fossil fuel combustion, biomass burning, and the oxidation of methane (CH_4_) and VOCs (Novelli et al., 1992). Anthropogenic sources of VOCs are largely due to industrial and vehicular emissions (Jaars et al., 2014), while biogenic VOCs are also naturally emitted (Jaars et al., 2016). Regional biomass burning, which includes household combustion for space heating and cooking, agricultural waste burning, and open biomass burning (wild fires), is a significant source of CO, NO_*x*_, and VOCs (Macdonald et al., 2011; Crutzen and Andreae, 1990; Galanter et al., 2000; Simpson et al., 2011) in southern Africa. In addition, stratospheric intrusions of O_3_-rich air to the free troposphere can also lead to elevated tropospheric O_3_ concentrations (Diab et al., 1996, 2004). O_3_ production from natural precursor sources, the long-range transport of O_3_, and the injections from stratospheric O_3_ contribute to background O_3_ levels, which is beyond the control of regulators (Lin et al., 2012).

Since O_3_ concentrations are regulated in South Africa, O_3_ monitoring is carried out across South Africa through a network of air quality monitoring stations established mainly by provincial governments, local municipalities, and industries (http://www.saaqis.org.za, last access: 30 November 2017). High O_3_ concentrations are observed in many areas within the interior of South Africa, which exceed the South African standard O_3_ limit, i.e. an 8 h moving average of 61 ppb (e.g. Laakso et al., 2013). These exceedances can be attributed to high anthropogenic emissions of NO_*x*_ and VOCs in dense urban and industrial areas (Jaars et al., 2014), regional biomass burning (Lourens et al., 2011), and O_3_-conducive meteorological conditions (e.g. sunlight). Since O_3_ is a secondary pollutant, high levels of O_3_ can also be found in rural areas downwind of city centres and industrial areas. In order for South Africa to develop an effective management plan to reduce O_3_ concentrations by controlling NO_*x*_ and VOC emissions, it is important to determine whether a region is NO_*x*_ or VOC limited. However, O_3_ production has a complex and non-linear dependence on precursor emissions (e.g. NRC, 1991), which makes its atmospheric levels difficult to control (Holloway and Wayne, 2010). Under VOC-limited conditions, O_3_ concentrations increase with increasing VOCs, while a region is considered NO_*x*_ limited when O_3_ production increases with increasing NO_*x*_ concentrations. Results from a photochemical box model study in South Africa, for instance, revealed that the Johannesburg-Pretoria megacity is within a VOC-limited regime (Lourens et al., 2016). VOC reductions would, therefore, be most effective in reducing O_3_, while NO_*x*_ controls without VOC controls may lead to O_3_ increases. In general, it is considered that O_3_ formations in regions close to anthropogenic sources are VOC limited, while rural areas distant from source regions are NO_*x*_ limited (Sillman, 1999).

Previous assessments of tropospheric O_3_ over continental South Africa have focused on surface O_3_ (Venter et al., 2012; Laakso et al., 2012; Lourens et al., 2011; Josipovic et al., 2010; Zunckel et al., 2004), as well as free tropospheric O_3_ based on soundings and aircraft observations (Diab et al., 1996, 2004; Thompson, 1996; Swap et al., 2003). Two major field campaigns (SAFARI-92 and SAFARI 2000) were conducted to improve the understanding of the effects of regional biomass burning emissions on O_3_ over southern Africa. These studies indicated a late winter-early spring (August and September) maximum over the region that was mainly attributed to increased regional open biomass burning during this period, while Lourens et al. (2011) also attributed higher O_3_ concentrations in spring in the Mpumalanga Highveld to increased regional open biomass burning. A more recent study demonstrated that NO_x_ strongly affects O_3_ levels in the Highveld, especially in winter and spring (Balashov et al., 2014). A regional photochemical modelling study (Zunckel et al., 2006) has attempted to explain surface O_3_ variability, which found no dominant source(s) of elevated O_3_ levels.

The aim of the current study is to provide an up-to-date assessment of the seasonal and diurnal variations in surface O_3_ concentrations over continental South Africa, as well as to identify local and regional sources of precursors contributing to surface O_3_. Another objective is to use available ambient data to qualitatively assess whether O_3_ formation is NO_x_ or VOC limited in different environments. An understanding of the key precursors that control surface O_3_ production is critical for the development of an effective O_3_ control strategy.

## 2 Methodology

### 2.1 Study area and measurement stations

**Figure 1.**
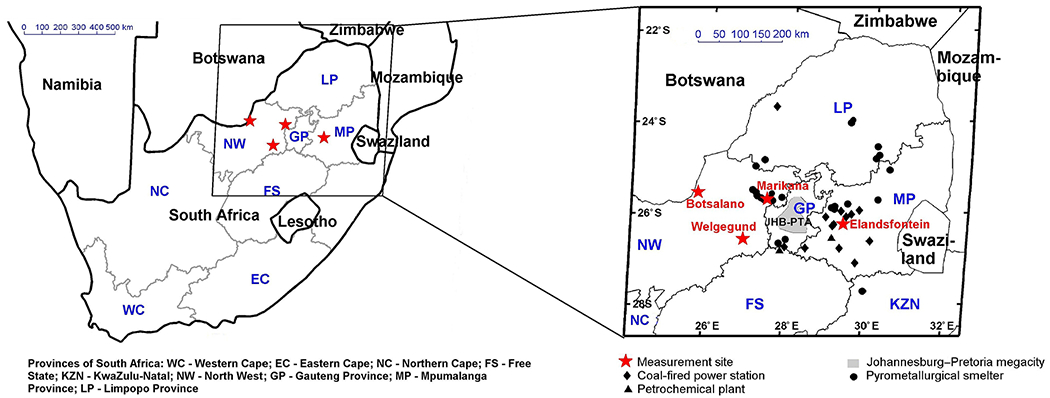
Location of the four measurement sites in South Africa.

Continuous in situ O_3_ measurements obtained from four research stations in the north-eastern interior of South Africa, indicated in Fig. 1, which include Botsalano (25.54° S, 25.75° E, 1420ma.s.l.), Marikana (25.70° S, 27.48° E, 1170ma.s.l.), Welgegund (26.57° S, 26.94° E, 1480 m a.s.l.), and Elandsfontein (26.25° S, 29.42° E, 1750ma.s.l.), were analysed. This region is the largest industrial (indicated by major point sources in Fig. 1) area in South Africa, with substantial gaseous and particulate emissions from numerous industries, domestic fuel burning, and vehicles (Lourens et al., 2012, 2011), while the Johannesburg–Pretoria megacity is also located in this area (Fig. 1). A combination of meteorology and anthropogenic activities has amplified the pollution levels within the region. The seasons in South Africa correspond to typical austral seasons, i.e. winter from June to August, spring from September to November, summer from December to February and autumn from March to May. The climate is semi-arid with an annual average precipitation of approximately 400 to 500 mm (Klopper et al., 2006; Dyson et al., 2015), although there is considerable inter-annual variability associated with the El Niño–Southern Oscillation (ENSO) phenomenon. Precipitation in the north-eastern interior occurs mostly during the austral summer, from October to March, whereas the region is characterized by a distinct cold and dry season from May to September, i.e. late autumn to mid-spring, during which almost no precipitation occurs. During this period, the formation of several inversion layers is present in the region, which limits the vertical dilution of air pollution, while more pronounced anticyclonic recirculation of air masses also occurs. This synoptic-scale meteorological environment leads to an accumulation of pollutants in the lower troposphere in this region, which can be transported for several days (Tyson and Preston-Whyte, 2000; Garstang et al., 1996). The SAFARI-92 and SAFARI 2000 campaigns indicated that locations in southern Africa, thousands of kilometres apart, are linked through regional anticyclonic circulation (Swap et al., 2003).

#### 2.1.1 Botsalano

The Botsalano measurement site is situated in a game reserve in the North West Province of South Africa, which is considered to be representative of regional background air. The surrounding vegetation is typical of a savannah biome, consisting of grasslands with scattered shrubs and trees (Laakso et al., 2008). The area is quite sparsely populated and has no local anthropogenic pollution sources (Laakso et al., 2008; Vakkari et al., 2013). The western Bushveld Igneous Complex, where numerous platinum, base metal, vanadium, and chromium mining–smelting industries are situated, is the largest regional anthropogenic pollution source, with the Rustenburg area located approximately 150 km to the east. Botsalano is also occasionally impacted by plumes passing over the industrialized Mpumalanga Highveld and the Johannesburg-Pretoria megacity (Laakso et al., 2008; Vakkari et al., 2011). In addition, the site is influenced by seasonal regional savannah wildfires during the dry period (Laakso et al., 2008; Vakkari et al., 2011; Mafusire et al., 2016). Measurements were conducted from 20 July 2006 until 5 February 2008 (Laakso et al., 2008; Vakkari et al., 2011, 2013).

#### 2.1.2 Marikana

The Marikana measurement site is located within the western Bushveld Igneous Complex, which is a densely populated and highly industrialized region, where mining and smelting are the predominant industrial activities. Marikana is a small mining town located approximately 30 km east of Rustenburg and approximately 100 km north-west of Johannesburg. The measurement site is located in the midst of a residential area, comprising low-cost housing settlements and municipal buildings (Hirsikko et al., 2012; Venter et al., 2012). Anthropogenic emissions from household combustion, traffic, and industry in the wider region have a strong influence on the measurement site (Venter et al., 2012). Data were collected from 8 February 2008 to 16 May 2010 and have been previously used in other studies (Venter et al., 2012; Vakkari et al., 2013; Petäjä et al., 2013; Hirsikko et al., 2012, 2013).

#### 2.1.3 Welgegund

This measurement site is approximately 100 km west of Johannesburg and is located on a commercial arable and pastoral farm. The station is surrounded by grassland savannah (Jaars et al., 2016). The station can be considered a regionally representative background site with few local anthropogenic sources. Air masses arriving at Welgegund from the west reflect a relatively clean regional background. However, the site is, similar to the Botsalano station, at times impacted by polluted air masses that are advected over major anthropogenic source regions in the interior of South Africa, which include the western Bushveld Igneous Complex, the Johannesburg–Pretoria megacity, the Mpumalanga Highveld, and the Vaal Triangle (Tiitta et al., 2014; Jaars et al., 2016; Venter et al., 2017). In addition, Welgegund is also affected by regional savannah and grassland fires that are common in the dry season (Vakkari et al., 2014). The atmospheric measurement station has been operating at Welgegund since 20 May 2010, with data measured up until 31 December 2015 utilized in this study.

#### 2.1.4 Elandsfontein

Elandsfontein is an ambient air quality monitoring station operated by Eskom, the national electricity supply company, primarily for legislative compliance purposes. This station was upgraded and co-managed by researchers during the EUCAARI project (Laakso et al., 2012). The Elandsfontein station is located within the industrialized Mpumalanga Highveld at the top of a hill approximately 200 km east of Johannesburg and 45 km south-south-east of eMalahleni (previously known as Witbank), which is a coal mining area (Laakso et al., 2012). The site is influenced by several emission sources, such as coal mines, coal-fired power-generating stations, a large petrochemical plant, and traffic emissions. Metallurgical smelters to the north also frequently impact the site (Laakso et al., 2012). The Elandsfontein dataset covers the period 11 February 2009 until 31 December 2010 during the EUCAARI campaign (Laakso et al., 2012).

### 2.2 Measurements

A comprehensive dataset of continuous measurements of surface aerosols, trace gases, and meteorological parameters has been acquired through these four measurement sites (Laakso et al., 2008, 2012; Vakkari et al., 2011, 2013; Venter et al., 2012; Petäjä et al., 2013). In particular, measurements of ozone (O_3_), nitric oxide (NO), nitrogen dioxide (NO_2_), and carbon monoxide (CO), as well as meteorological parameters, such as temperature (°C) and relative humidity (RH, %), were used in this study. Note that Botsalano, Marikana, and Welgegund measurements were obtained with the same mobile station (first located at Botsalano, then relocated to Marikana and thereafter permanently positioned at Welgegund), while Elandsfontein measurements were conducted with a routine monitoring station. O_3_ concentrations at Welgegund, Botsalano, and Marikana research stations were measured using the Environment SA 41M O_3_ analyser, while a Monitor Europe ML9810B O_3_ analyser was utilized at Elandsfontein. CO concentrations were determined at Welgegund, Botsalano, and Marikana with a Horiba APMA-360 analyser, while CO was not measured at Elandsfontein. NO_*x*_ (NO + NO_2_) concentrations were determined with a Teledyne 200AU NO/NO_x_ analyser at Welgegund, Botsalano and Marikana, whereas a Thermo Electron 42i NO-NO2-NOx analyser was used at Elandsfontein. Temperature and RH were measured with a Rotronic MP 101A instrument at all the sites.

Data quality at these four measurement sites was ensured through regular visits to the sites, during which instrument maintenance and calibrations were performed. The data collected from these four stations were subjected to detailed cleaning (e.g. excluding measurements recorded during power interruptions, electronic malfunctions, calibrations, and maintenance) and the verification of data quality procedures (e.g. corrections were made to data according to in situ calibrations and flow checks). Therefore, the datasets collected at all four measurement sites are considered to represent high-quality, high-resolution measurements as indicated by other papers (Laakso et al., 2008, 2012; Petäjä et al., 2013; Venter et al., 2012; 2011; Vakkari et al., 2013). Detailed descriptions of the data post-processing procedures were presented by Laakso et al. (2008) and Venter et al. (2012). The data were available as 15 min averages and all plots using local time (LT) refer to local South African time, which is UTC + 2.

In order to obtain a representative spatial coverage of continental South Africa, O_3_ data from an additional 54 ambient monitoring sites were selected. These included O_3_ measurements from 18 routine monitoring station measurements (SAAQIS) for the period from January 2012 to December 2014 (downloaded from the JOIN web interface https://join.fz-juelich.de, last access: 15 July 2017; Schultz et al., 2017) and 36 passive sampling sites located in the north-eastern interior of South Africa where monthly O_3_ concentrations were determined for 2 years from 2006 to 2007 (Josipovic, 2009). Spatial analyses were conducted with a geographic information system mapping tool (ArcGIS software), which used ordinary kriging to interpolate the O_3_ concentrations measured at the 58 sites in order to build the spatial distribution. The interpolation method involved making an 80/20 % split of the data (80 % for model development, 20 % for evaluation), in which 20 % was used to calculate the root-mean-square error (RMSE = 0.2804331). Optimal model parameters were selected using an iterative process and evaluated on the basis of the best performance statistics obtained (reported in the ArcGIS kriging output), with particular emphasis on minimizing the RMSE. The extent of area was 23.00154974 (top), −29.03070026 (bottom), 25.74238974 (left), and 32.85246366 (right).

### 2.3 Air mass history

Individual hourly 4-day back trajectories for air masses arriving at an arrival height of 100 m above ground level were calculated for the entire measurement period at each monitoring site, using HYSPLIT 4.8 (Hybrid Single-Particle Lagrangian Integrated Trajectory model) (Stein et al., 2015; Draxler and Hess, 1998). The model was run with the GDAS meteorological archive produced by the US National Weather Service’s National Centre for Environmental Prediction (NCEP) and archived by ARL (Air Resources Laboratory, 2017). Overlay back trajectory maps were generated by superimposing individual back trajectories onto a southern African map divided into 0.5° × 0.5° grid cells. In addition, source maps were compiled by assigning each grid cell with a mean measured O_3_ and CO concentration associated with trajectories passing over that cell, similar to previous methods (Vakkari et al., 2011, 2013; Tiitta et al., 2014). A minimum of 10 trajectories per cell were required for the statistical reliability.

### 2.4 Modelling instantaneous production rate of O_3_

**Figure A1.**
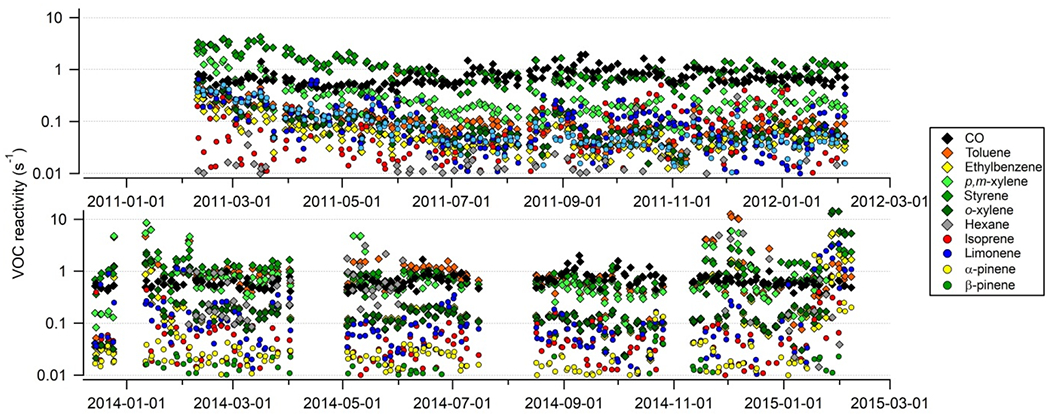
Individual VOC reactivity time series. In the calculation of instantaneous O_3_ production (*P*(O_3_)), CO was treated as a VOC.

The only speciated VOC dataset available and published in South Africa exists for Welgegund (Jaars et al., 2016, 2014), which could be used to model instantaneous O_3_ production at this site. The concentration of these biogenic and anthropogenic VOCs was obtained from grab samples taken between 11:00 and 13:00 LT over the course of two extensive field campaigns conducted from February 2011 to February 2012 and from December 2013 to February 2015. During this time, six trace gases, 19 biogenic VOCs, and 20 anthropogenic VOCs, including 13 aromatic and seven aliphatic compounds were measured. The VOC reactivity was calculated from the respective rate coefficients of each VOC with ^•^OH radicals obtained from chemical kinetic databases such as JPL, NIST, and the MCM (e.g. Jaars et al., 2014) to estimate ozone production at 11:00 LT at Welgegund. Specifically, each VOC reactivity was then summed to obtain the total VOC reactivity for each measurement, i.e. VOC reactivity = Σ*k*_1*i*_ [VOC]_*I*_. The major contributors to VOC reactivity are depicted in Fig. A1 and include, in approximate order of contribution, *o*-xylene, CO, styrene, *p*,*m*-xylene, toluene, ethylbenzene limonene, isoprene, *α*-pinene, *β*-pinene, and hexane. Of note, key compounds such as methane are not included, which could contribute to VOC reactivity, and therefore this VOC reactivity can only be a lower estimate. However, if a global ambient concentration of 1.85 ppm and a rate of oxidation by ^•^OH radicals of 6.68 × 10^−15^ cm^3^ molec^−1^ s^−1^ are assumed (Srinivasan et al., 2005), a VOC reactivity of 0.3 s^−1^ would be obtained and would therefore account for a small increase in the VOC reactivity calculated in Figs. A1 and 10.

A mathematical box model was applied to model O_3_ production as a function of VOC reactivity and NO2 concentrations. This model involves three steps, i.e. (1) the estimation of HO_*x*_ (sum of ^•^OH and ${\text{HO}}_{2}^{•}$ radicals) production, (2) the estimation of the ^•^OH radical concentration, and (3) the calculation for O_3_ production (Murphy et al., 2006; Geddes et al., 2009). The VOC concentrations are the limiting factor in the ability to model O_3_ production at Welgegund since only data for the 11:00 to 13:00LT grab samples were available (Fig. A1). Therefore, the model approach does not coincide with peak O_3_ typically observed around 14:00 to 15:00LT and therefore likely represents a lower estimate.

The production rate of HO_*x*_ (*P*(HO_*x*_)) depends on the photolysis rate of O_3_ (*J*_O_3__), concentration of O_3_, and vapour pressure of water (Jaegld et al., 2001). The photolysis rate proposed for the Southern Hemisphere, i.e. *J*_O__3_ = 3 × 10^−5^ s^−1^ (Wilson, 2015), was used, from which P(HO_x_) was calculated as follows:

$$P\;({\text{HO}}_{x})=2J_{\text{O}3}k_{\text{O}3}\left[O_{3}\right]\left[{\text{H}}_{2}\text{O}\right]$$

and estimated to be 6.09 × 10^6^ molec cm^−3^ s^−1^ or 0.89 ppbv h^−1^ (calculated for a campaign O_3_ average of 41 ppbv and a campaign RH average of 42% at 11:00LT each day) at STP. The *P*(HO_*x*_) at Welgegund is approximately a factor of 2 lower compared to other reported urban P(HO_x_) values (Geddes et al., 2009). The factors and reactions that affect [^•^OH] include

- linear dependency between ^•^OH and NO_*x*_ due to the reaction NO+HO_2_→^•^OH+NO_2_, until ^•^OH begins to react with elevated NO_2_ concentrations to form HNO_3_ (OH+NO_2_ + M→HNO_3_ +M);
- *P*(HO_*x*_) affected by solar irradiance, temperature, O_3_ concentrations, and humidity; and
- partitioning of HO_*x*_ among RO_2_, HO_2_, and OH.

[•OH] was calculated at 11:00LT each day as follows:

$$A=k_{5eff}{\left(\frac{\text{VOC}\;\text{reactivity}}{k_{2eff}\left[\text{NO}\right]}\right)}^{2}$$

$$B=k_{4}\left[{\text{NO}}_{2}\right]+\alpha \times \text{VOC}\;\text{reactivity}$$

$$C=P\left({\text{HO}}_{x}\right)$$

$$\left[\text{OH}\right]=\frac{-B+\sqrt{B^{2}+24C+A}}{12\times A}$$

The instantaneous production rate of O_3_, P (O_3_), could then be calculated as a function of NO_2_ levels and VOC reactivity. A set of reactions used to derive the equations that describe the dependence of the ^•^OH, peroxy radicals $\left({\text{HO}}_{2}^{•}+{\text{RO}}_{2}^{•}\right)$, and P (O_3_) on NO_*x*_ is given by Murphy et al. (2006), which presents the following equation to calculate P (O_3_):

$$\begin{array}{l} P\;({\text{O}}_{3})=k_{2eff}\left[{\text{HO}}_{2}+{\text{RO}}_{2}\right]\left[\text{NO}\right]\\ =2\times \text{VOC}\;\text{Reactivity}\times \left[\text{OH}\right], \end{array}$$

where *k*_2eff_ is the effective rate constant of NO oxidation by peroxy radicals (chain propagation and termination reactions in the production of O_3_). The values of the rate constants and other parameters used as input parameters to solve the equation above can be found in Murphy et al. (2006) and Geddes et al. (2009).

## 3 Results and discussion

### 3.1 Temporal variation in O_3_

**Figure 2.**
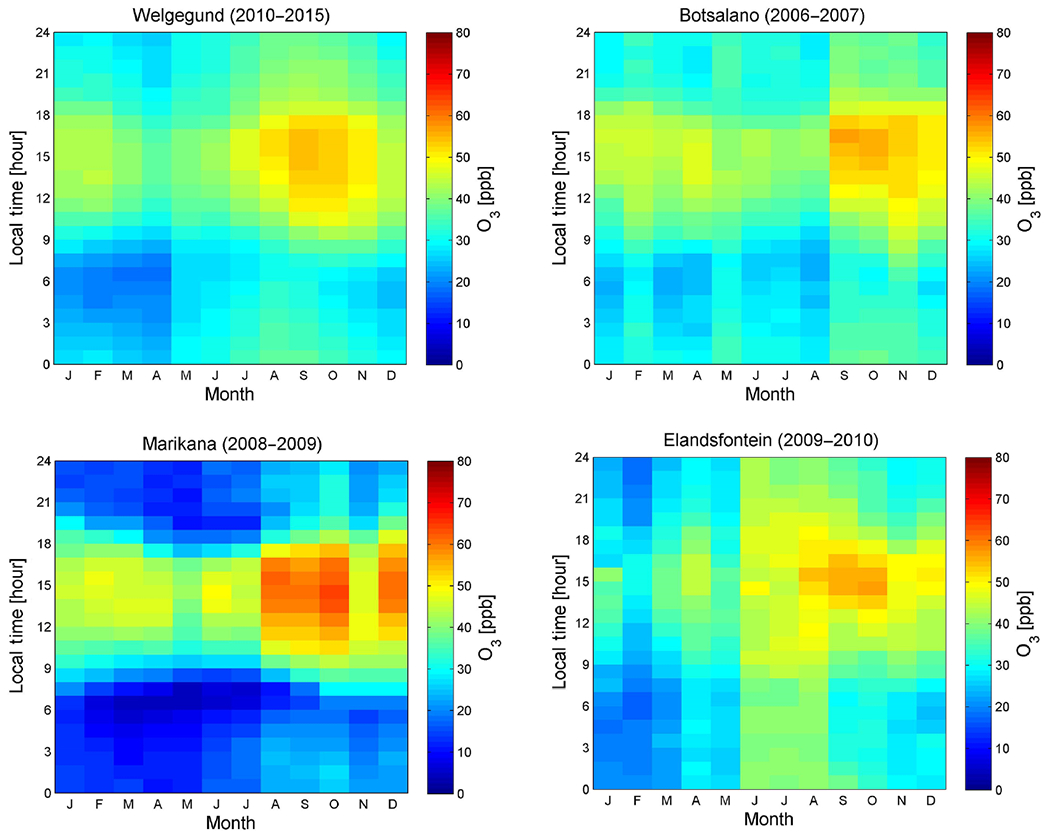
Seasonal and diurnal variation in median O_3_ concentrations at Welgegund, Botsalano, Marikana, and Elandsfontein. The O_3_ measurement periods varied among sites, which combined spanned a period from July 2006 to December 2015.

**Figure A2.**
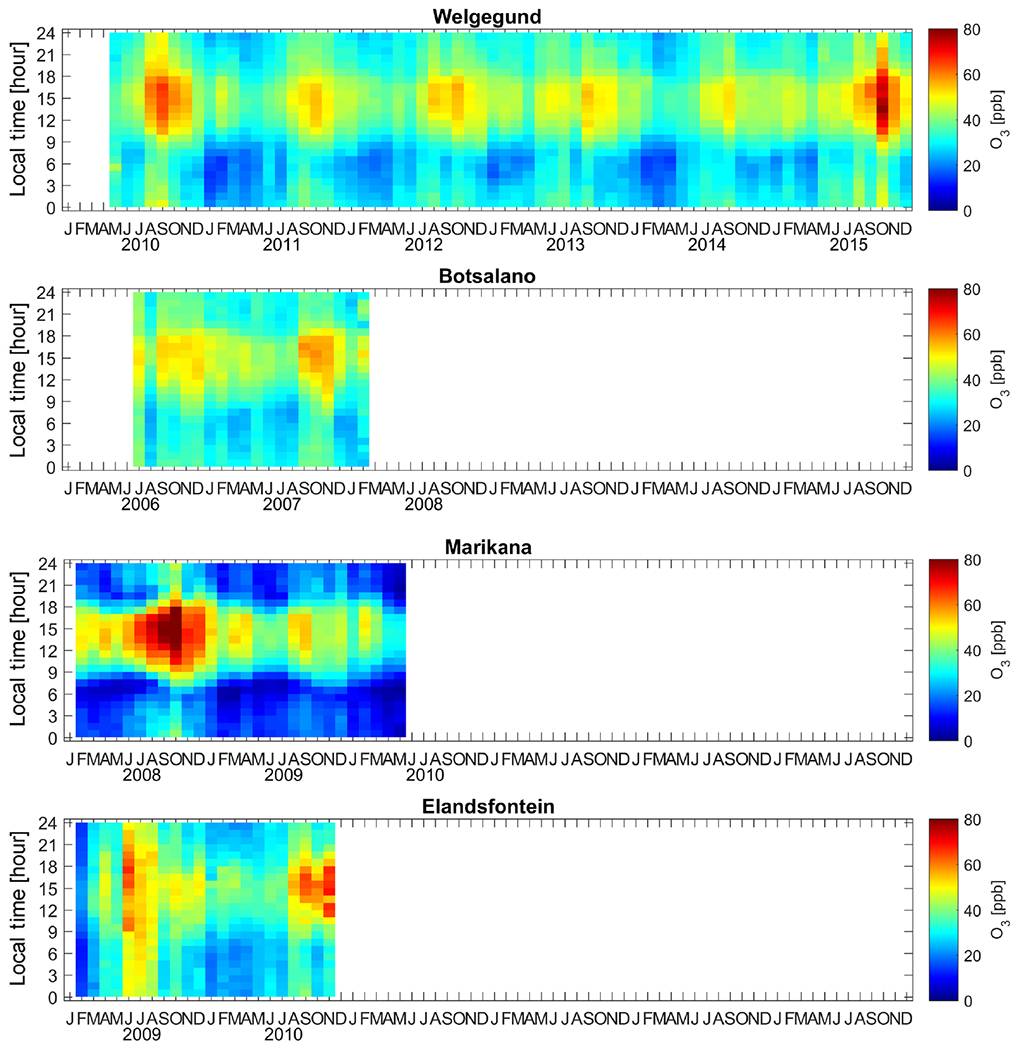
Time series of monthly median O_3_ concentrations for each hour of the day at the four sites.

**Figure A3.**
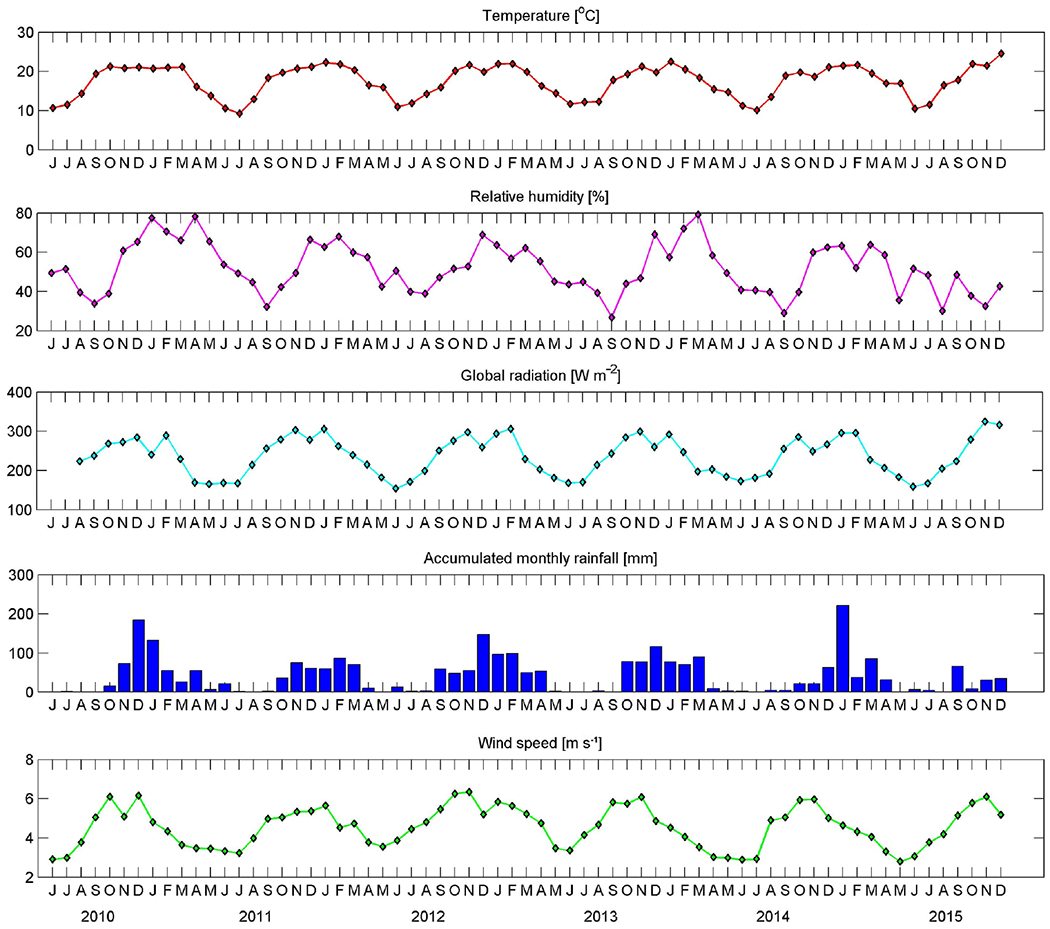
Monthly averages of meteorological parameters at Welgegund to show typical seasonal patterns in continental South Africa. In the case of rainfall, the total monthly rainfall values are shown.

In Fig. 2, the monthly and diurnal variations for O_3_ concentrations measured at the four sites in this study are presented (time series plotted in Fig. A2). Although there is some variability among the sites, monthly O_3_ concentrations show a well-defined seasonal variation at all four sites, with maximum concentrations occurring in late winter and spring (August to November), which is expected for the South African interior as indicated above and previously reported (Zunckel et al., 2004; Diab et al., 2004). In Fig. A3 monthly averages of meteorological parameters and total monthly rainfall for Welgegund are presented to indicate typical seasonal meteorological patterns for continental South Africa. These O_3_ peaks in continental South Africa generally point to two major contributors of O_3_ precursors, i.e. open biomass burning (wild fires) (Vakkari et al., 2014) and increased low-level anthropogenic emissions, e.g. increased household combustion for space heating and cooking (Oltmans et al., 2013; Lourens et al., 2011). In addition to the seasonal patterns of O_3_ precursor species, during the dry winter months, synoptic-scale recirculation is more predominant and inversion layers are more pronounced, while precipitation is minimal (e.g. Tyson and Preston-Whyte, 2000). These changes in meteorology result in the build-up of precursor species that reach a maximum in August–September when photochemical activity starts to increase. The diurnal concentration profiles of O_3_ at the four locations follow the typical photochemical cycle, i.e. increasing during daytime in response to maximum photochemical production and decreasing during the night-time due to titration with NO. O_3_ levels peaked from midday to afternoon, with a maximum at approximately 15:00 (LT, UTC+2). From Fig. 2, it is also evident that night-time titration of O_3_ at Marikana is more pronounced, as indicated by the largest difference between daytime and night-time O_3_ concentrations in comparison to the other sites, especially compared to Elandsfontein where night-time concentrations of O_3_ remain relatively high in winter.

### 3.2 Spatial distribution of O_3_ in continental South Africa

**Figure 3.**
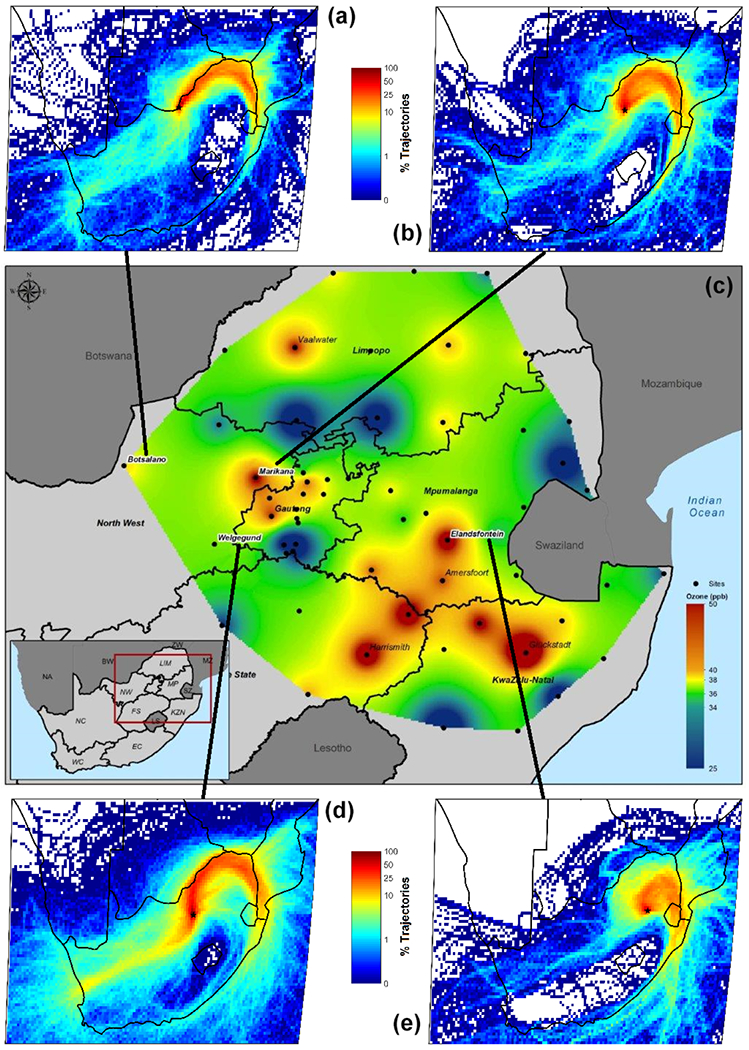
The main (central) map indicating spatial distribution of mean surface O_3_ levels during springtime over the north-eastern interior of southern Africa ranging between 23.00 and 29.03° S and between 25.74 and 32.85° E. The data for all sites were averaged for years when the ENSO cycle was not present (by examining sea surface temperature anomalies in the Niño 3.4 region). Black dots indicate the sampling sites. The smaller maps (top and bottom) indicate 96 h overlay back trajectory maps for the four main study sites, over the corresponding springtime periods.

Figure 3 depicts the spatial pattern of mean surface O_3_ concentrations over continental South Africa during springtime (September–October–November), when O_3_ is usually at a maximum, as indicated above. Also presented in Fig. 3, are 96 h overlay back trajectory maps for the four main study sites over the corresponding springtime periods. The mean O_3_ concentration over continental South Africa ranged from 20 to 60 ppb during spring. From Fig. 3, it can be seen that O_3_ concentrations at the industrial sites Marikana and Elandsfontein were higher than O_3_ levels at Botsalano and Welgegund. As mentioned previously, Elandsfontein is located within the industrialized Mpumalanga Highveld with numerous large point sources of O_3_ precursor species. It is also evident from Fig. 3 that rural measurement sites downwind from Elandsfontein, such as Amersfoort, Harrismith, and Glückstadt had significantly higher O_3_ concentrations, which can be attributed to the formation of O_3_ during the transport of precursor species from source regions. Lourens et al. (2011) indicated that higher O_3_ concentrations were associated with sites positioned in more rural areas in the Mpumalanga Highveld. Venter et al. (2012) attributed high O_3_ concentrations at Marikana, which exceeded South African standard limits on a number of occasions, to the influence of local household combustion for cooking and space heating, as well as to regional air masses with high O_3_ precursor concentrations. Higher O_3_ concentrations were also measured in the northwestern parts of Gauteng, at sites situated within close proximity to the Johannesburg–Pretoria megacity, while the rural Vaalwater site in the north also has significantly higher O_3_ levels. From Fig. 3, it is evident that O_3_ can be considered a regional problem, with O_3_ concentrations being relatively high across continental South Africa during spring. Figure 3 also clearly indicates that the four research sites where surface O_3_ was assessed in this study are representative of continental South Africa.

### 3.3 Comparison with international sites

**Figure 4.**
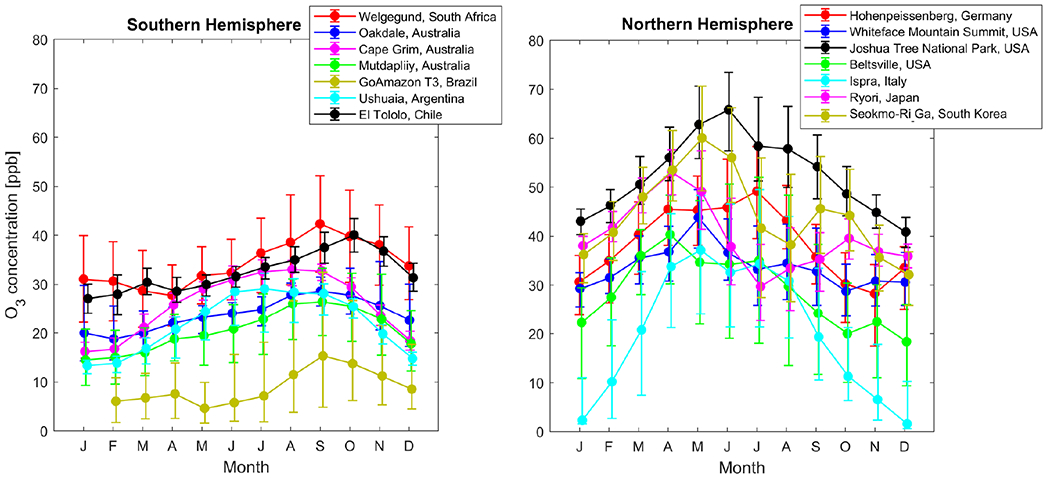
Seasonal cycle of O_3_ at rural sites in other parts of the world. The dots indicate monthly median (50th percentile) and the upper and lower limits the 25th and 75th percentiles, respectively, for monthly O_3_ concentrations. The data are averaged from May 2010 to December 2014, except in a few instances in which 2014 data were not available.

In an effort to contextualize the O_3_ levels measured in this study, the monthly O_3_ concentrations measured at Welgegund were compared to monthly O_3_ levels measured at monitoring sites in other parts of the world (downloaded from the JOIN web interface https://join.fz-juelich.de; Schultz et al., 2017) as indicated in Fig. 4. Welgegund was used in the comparison since it had the most extensive data record, while the measurement time period considered was from May 2010 to December 2014. The seasonal O_3_ cycles observed at other sites in the Southern Hemisphere are comparable to the seasonal cycle at Welgegund, with slight variations in the time of year when O_3_ peaks, as indicated in Fig. 4. Cape Grim, Australia; GoAmazon T3 Manancapuru, Brazil; Ushuaia, Argentina; and Cerro Tololo, Chile, are regional background GAW (Global Atmosphere Watch) stations with O_3_ levels lower than the South African sites. However, the O_3_ concentrations at Cerro Tololo, Chile, are comparable to Welgegund. Oakdale, Australia, and Mutdapilly, Australia, are semi-rural and rural locations, which are influenced by urban and industrial pollution sources and also had lower O_3_ concentrations compared to Welgegund.

The northern hemispheric O_3_ peak over mid-latitude regions is similar to seasonal patterns in the Southern Hemisphere where a springtime O_3_ maximum is observed (i.e. Whiteface Mountain Summit, Beltsville, Ispra, Ryori, and Seokmo-Ri Ga). However, there are other sites in the Northern Hemisphere where a summer maximum is more evident (Vingarzan, 2004), i.e. Joshua Tree and Hohenpeissenberg. The discernible difference between the hemispheres is that the spring maximum in the Southern Hemisphere refers to maximum O_3_ concentrations in late winter and early spring, while in the Northern Hemisphere, it refers to a late spring and early summer O_3_ maximum (Cooper et al., 2014). The spring maximum in the Northern Hemisphere is associated with stratospheric intrusions (Zhang et al., 2014; Parrish et al., 2013), while the summer maximum is associated with photochemical O_3_ production from anthropogenic emissions of O_3_ precursors being at its highest (Logan, 1985; Chevalier et al., 2007). Maximum O_3_ concentrations at background sites in the United States and Europe are similar to values at Welgegund in spring with the exception of Joshua Tree National Park in the United States, which had significantly higher O_3_ levels. This is most likely due its high elevation and deep boundary layer (~ 4kma.s.l.) during spring and summer allowing free-tropospheric O_3_ to be more effectively mixed down to the surface (Cooper et al., 2014). Maximum O_3_ levels at the two sites in East Asia (Ryori and Seokmo-Ri Ga) were also generally higher than at Welgegund, especially at Seokmo-Ri Ga.

### 3.4 Sources contributing to surface O_3_ in continental South Africa

As indicated above (Sect. 3.1), the O_3_ peaks in continental South Africa usually reflect increased concentrations of precursor species from anthropogenic sources during winter, as well as the occurrence of regional open biomass burning in late winter and early spring. In addition, stratospheric O_3_ intrusions during spring (Lefohn et al., 2014) could also partially contribute to increased surface O_3_ levels.

#### 3.4.1 Anthropogenic and open biomass burning emissions

**Figure A4.**
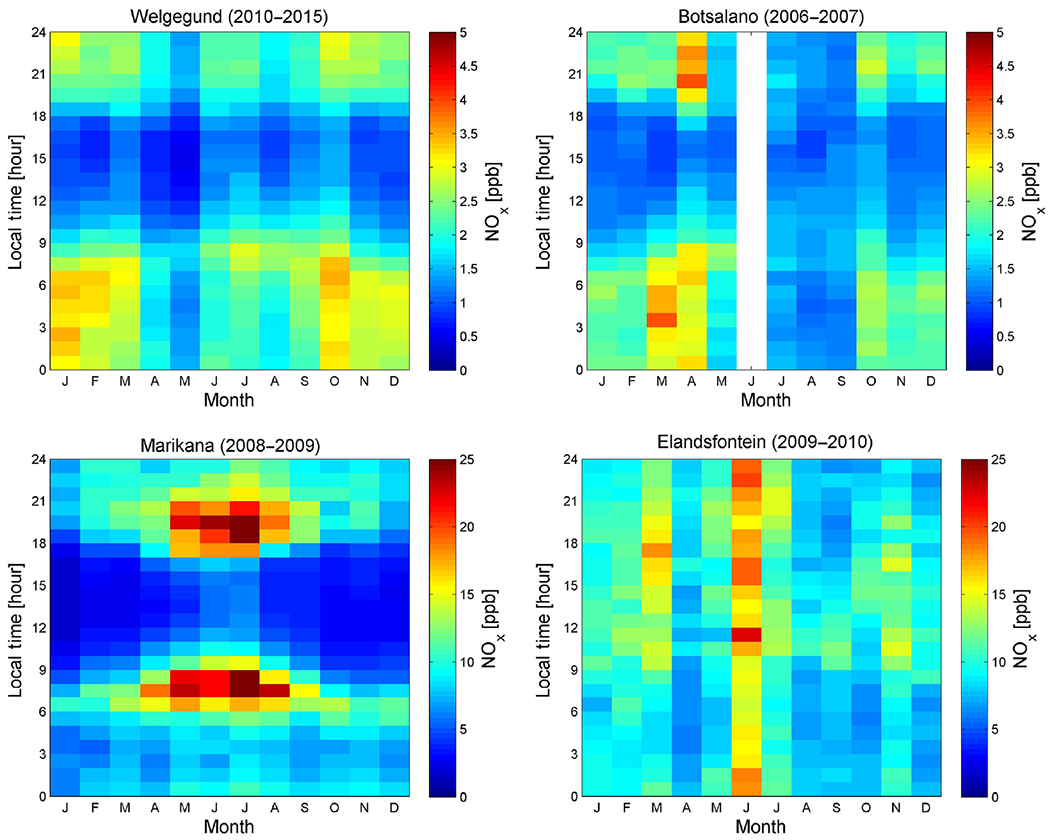
Seasonal and diurnal variation in NO_*x*_ at Welgegund, Botsalano, Marikana, and Elandsfontein (median values of NO_*x*_ concentration were used).

**Figure A5.**
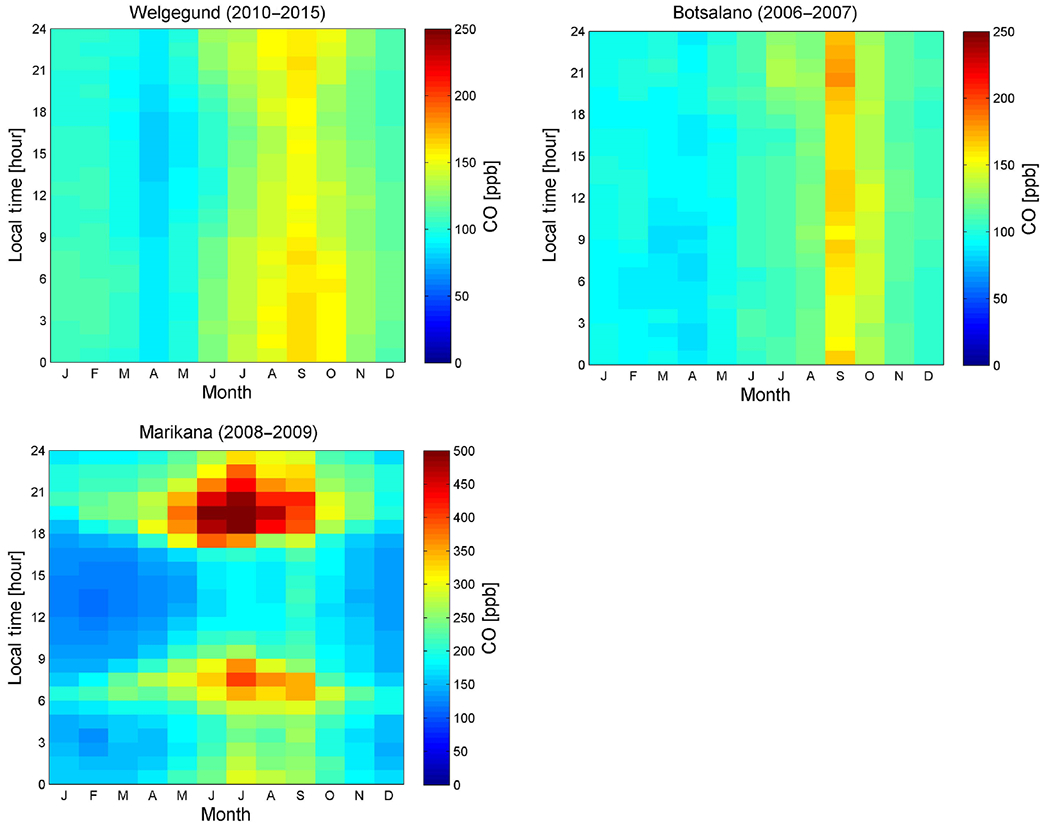
Seasonal and diurnal variation in CO at Welgegund, Botsalano, and Marikana (median values of CO concentration were used). Note that CO was not measured at Elandsfontein.

A comparison of the O_3_ seasonal cycles at background and polluted locations is useful for source attribution. From Fig. 2, it is evident that daytime O_3_ levels peaked at Elandsfontein, Marikana and Welgegund during late winter and spring (August to October), while O_3_ levels at Botsalano peaked later in the year during spring (September to November). This suggests that Elandsfontein, Marikana and Welgegund were influenced by increased levels of O_3_ precursors from anthropogenic and open biomass burning emissions (i.e. NO_*x*_ and CO indicated in Figs. A4 and A5, respectively – time series plotted in Figs. A7 and A8), while O_3_ levels at Botsalano were predominantly influenced by regional open biomass burning (Fig. A5). Although Welgegund and Botsalano are both background sites, Botsalano is more removed from anthropogenic source regions than Welgegund is (Sect. 2.1.3), which is therefore not directly influenced by the increased concentrations of O_3_ precursor species associated with anthropogenic emissions during winter. Daytime O_3_ concentrations were the highest at Marikana throughout most of the year, which indicates the influence of local and regional sources of O_3_ precursors at this site (Venter et al., 2012). In addition, a larger difference between O_3_ concentrations in summer and winter–spring is observed at Marikana compared to Welgegund and Botsalano, which can be attributed to local anthropogenic emissions (mainly household combustion) of O_3_ precursors at Marikana.

O_3_ concentrations at Elandsfontein were lower compared to the other three sites throughout the year, with the exception of the winter months (June to August). The major point sources at Elandsfontein include NO_*x*_ emissions from coal-fired power stations and are characterized by high-stack emissions, which are emitted above the low-level night-time inversion layers. During daytime, downwards mixing of these emitted species occurs, which results in daytime peaks of NO_x_ (as indicated in Fig. A4 and by Collett et al., 2010) and subsequent O_3_ titration. In contrast, Venter et al. (2012) indicated that, at Marikana, low-level emissions associated with household combustion for space heating and cooking were a significant source of O_3_ precursor species, i.e. NO_*x*_ and CO. The diurnal pattern of NO_*x*_ and CO (Figs. A4 and A5, respectively) at Marikana was characterized by bimodal peaks during the morning and evening, which resulted in increased O_3_ concentrations during daytime and night-time titration of O_3_, especially during winter. Therefore, the observed differences in night-time titration at Marikana and Elandsfontein can be attributed to different sources of O_3_ precursors, i.e. mainly low-level emissions (household combustion) at Marikana (Venter et al., 2012) compared to predominantly high-stack emissions at Elandsfontein (Collett et al., 2010). The higher O_3_ concentrations at Elandsfontein during winter are most likely attributed to the regional increase in O_3_ precursors.

**Figure 5.**
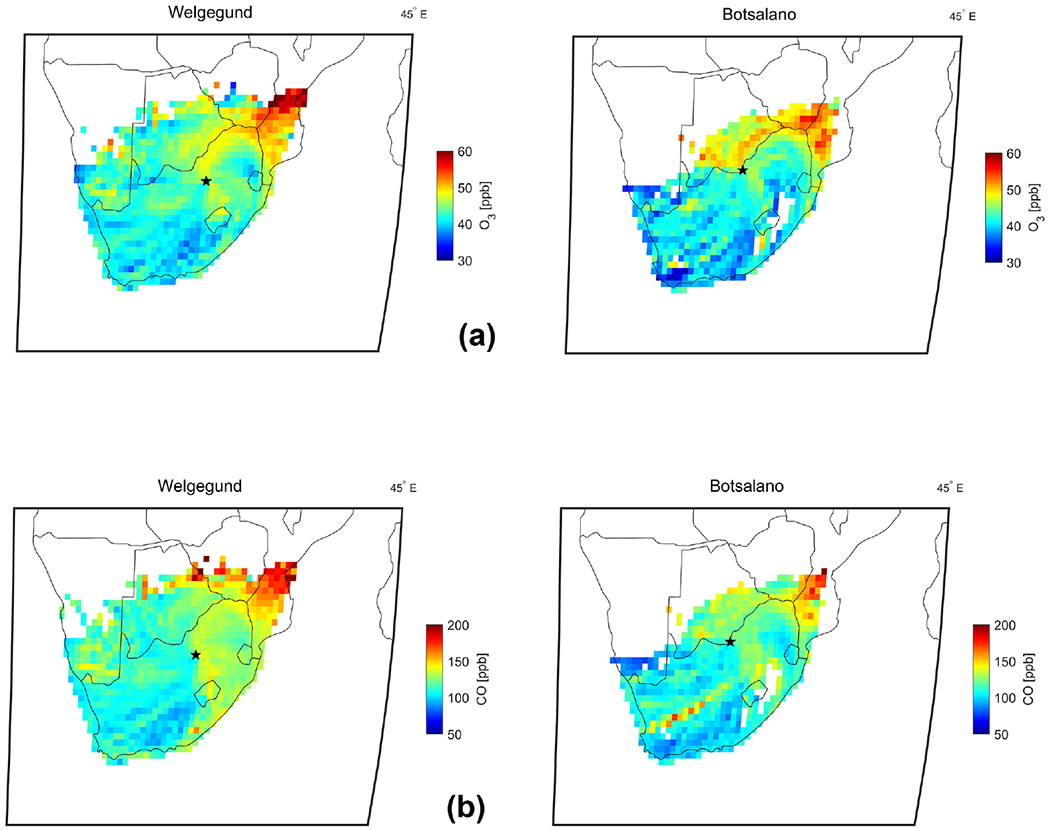
Source area maps of **(a)** O_3_ concentrations and **(b)** CO concentrations for the background sites Welgegund and Botsalano. The black star represents the measurement site and the colour of each pixel represents the mean concentration of the respective gas species. At least 10 observations per pixel are required.

**Figure 6.**
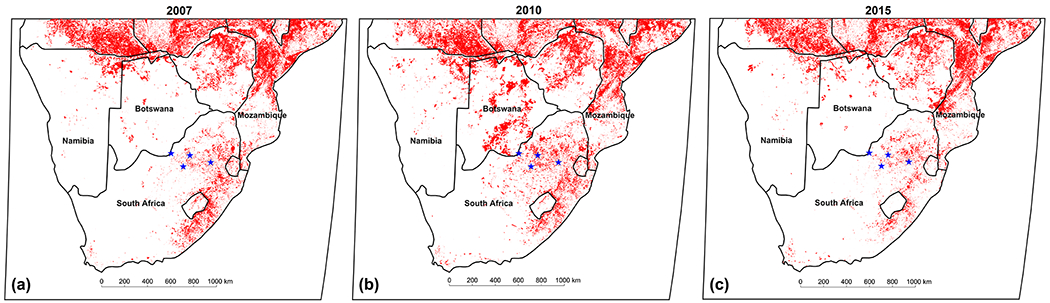
Spatial distribution of fires in 2007, 2010, and 2015 from the MODIS burnt area product. Blue stars indicate (from left to right) Botsalano, Welgegund, Marikana, and Elandsfontein.

The spring maximum O_3_ concentrations can be attributed to increases in widespread regional biomass burning in this region during this period (Vakkari et al., 2014; Lourens et al., 2011). Biomass burning has strong seasonality in southern Africa, extending from June to September (Galanter et al., 2000), and is an important source of O_3_ and its precursors during the dry season. In an effort to elucidate the influence of regional biomass burning on O_3_ concentrations in continental South Africa, source area maps of O_3_ were compiled by relating O_3_ concentrations measured with air mass history, which are presented in Fig. 5a. Source area maps were only generated for the background sites Welgegund and Botsalano since local sources at the industrial sites Elandsfontein and Marikana would obscure the influence of regional biomass burning. In addition, maps of spatial distribution of fires during 2007, 2010, and 2015 were compiled with the MODIS Collection 5 burnt area product (Roy et al., 2008, 2005, 2002) and are presented in Fig. 6.

The highest O_3_ concentrations measured at Welgegund and Marikana were associated with air masses passing over a sector north to north-east of these sites, i.e. southern and central Mozambique, southern Zimbabwe, and south-eastern Botswana. O_3_ concentrations associated with air masses passing over central and southern Mozambique were particularly high. In addition to O_3_ source maps, CO source maps were also compiled for Welgegund and Botsalano, as indicated in Fig. 5b. It is evident that the CO source maps indicated a similar pattern to that observed for O_3_, with the highest CO concentrations corresponding with the same regions where O_3_ levels are the highest. From the fire maps in Fig. 6, it can be observed that a large number of fires occur in the sector, associated with higher O_3_ and CO concentrations, with the fire map indicating, in particular, a high fire frequency occurring in central Mozambique. During 2007, more fires occurred in Botswana compared to the other two years, which is also reflected in the higher O_3_ levels measured at Botsalano during that year for air masses passing over this region. Open biomass burning is known to emit more CO than NO_*x*_, while CO also has a relatively long atmospheric lifetime (1 to 2 months; Kanakidou and Crutzen, 1999) compared to NO_*x*_ (6 to 24 h, Beirle et al., 2003) and VOCs (a few hours to a few weeks; Kanakidou and Crutzen, 1999) emitted from open biomass burning. Enhanced CO concentrations have been used previously to characterize the dispersion of biomass burning emissions over southern Africa (Mafusire et al., 2016). Therefore, the regional transport of CO and VOCs (and NO_*x*_ to a lesser extent) associated with biomass burning occurring from June to September in southern Africa can be considered an important source of surface O_3_ in continental South Africa (Fig. A5).

#### 3.4.2 Stratospheric O_3_

**Figure 7.**
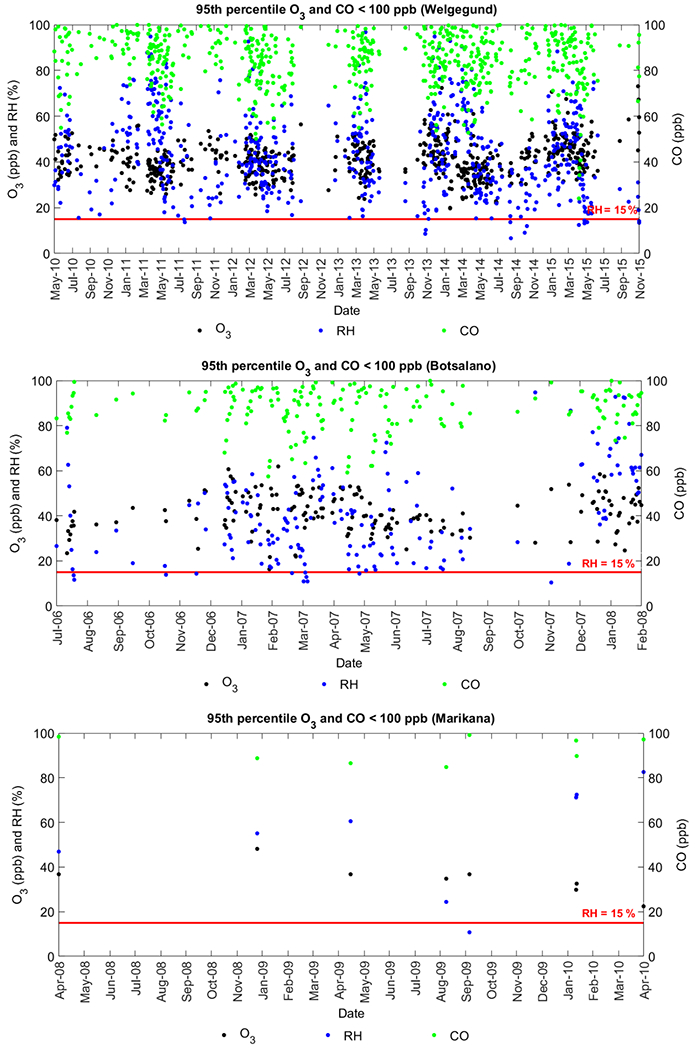
Simultaneous measurements of O_3_ (daily 95th percentile), CO (daily average ppb), and RH (daily average) from 07:00 to 18:00 LT at Welgegund, Botsalano, and Marikana.

Elevated levels of tropospheric O_3_ may also be caused by stratospheric intrusion of O_3_-rich air (Zhang et al., 2014; Parrish et al., 2013; Lin et al., 2012), especially on certain days during late winter and spring when O_3_ is the highest on the South African Highveld (Thompson et al., 2014). However, the importance of the stratospheric source over continental South Africa has not yet been specifically addressed. The assessment of meteorological fields and air quality data at high-elevation sites is required to determine the downward transport of stratospheric O_3_. Alternatively, stratospheric O_3_ intrusions can be estimated through concurrent in situ measurements of ground-level O_3_, CO, and humidity since stratospheric intrusions of O_3_ into the troposphere are characterized by elevated levels of O_3_, high potential vorticity, low levels of CO, and low water vapour (Stauffer et al., 2017; Thompson et al., 2015, 2014). Thompson et al. (2015) defined low CO as 80 to 110ppbv, while low RH is considered < 15 %. In Fig. 7, the 95th percentile O_3_ levels (indicative of “high O_3_”) corresponding to low daily average CO concentrations (< 100 ppb) are presented together with the daily average RH. Only daytime data from 07:00 to 18:00 LT were considered in order to exclude the influence of night-time titration. From Fig. 7, it is evident that very few days complied with the criteria indicative of stratospheric O_3_ intrusion, i.e. high O_3_, low CO, and low RH, which indicates a very small influence of stratospheric intrusion on surface O_3_ levels. However, it must be noted that the attempt in this study to relate surface O_3_ to stratospheric intrusions is a simplified qualitative assessment and more quantitative detection methods should be applied to understand the influence of stratospheric intrusions on surface O_3_ for this region.

### 3.5 Insights into the O_3_ production regime

The relationship among O_3_, NO_*x*_, and CO was used as an indicator to infer the O_3_ production regime at Welgegund, Botsalano, and Marikana (no CO measurements were conducted at Elandsfontein as indicated above) since no continuous VOC measurements were conducted at each of these sites. However, as indicated in Sect. 2.4, a 2-year VOC dataset was available for Welgegund (Jaars et al., 2016,^2014^), which was used to calculate the instantaneous production rate of O_3_ as a function of NO_2_ levels and VOC reactivity (Geddes et al., 2009; Murphy et al., 2006).

#### 3.5.1 The relationship among NO_*x*_, CO, and O_3_

**Figure 8.**
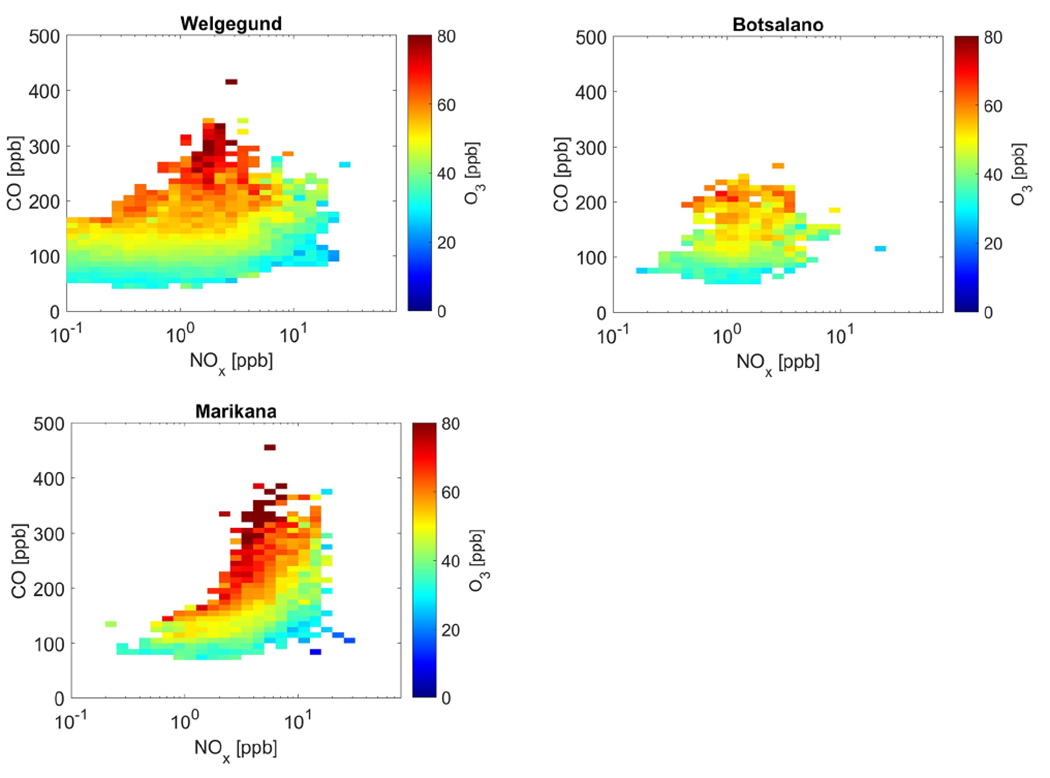
Mean O_3_ concentration averaged for NO_*x*_ and CO bins. Measurements were only taken from 11:00 to 17:00 LT when photochemical production of O_3_ was at a maximum.

**Figure A6.**
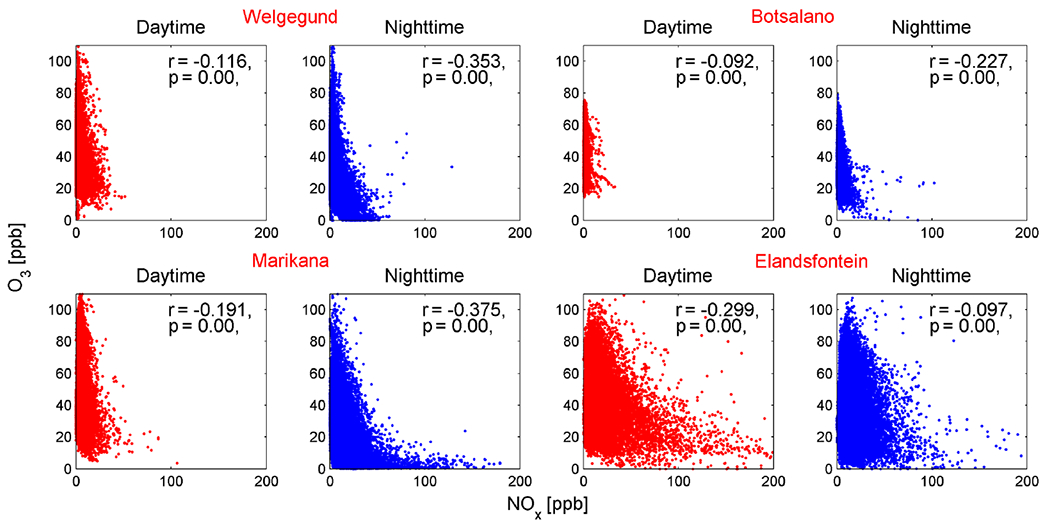
Scatter plots of O_3_ vs. NO_*x*_ for daytime (09:00 to 16:52 LT), and night-time (17:00 to 08:52 LT) at Welgegund, Botsalano, Marikana, and Elandsfontein. The correlation coefficient (*r*) has a significance level of *p* < 10^−10^, which means that *r* is statistically significant (*p* < 0.01).

**Figure A7.**
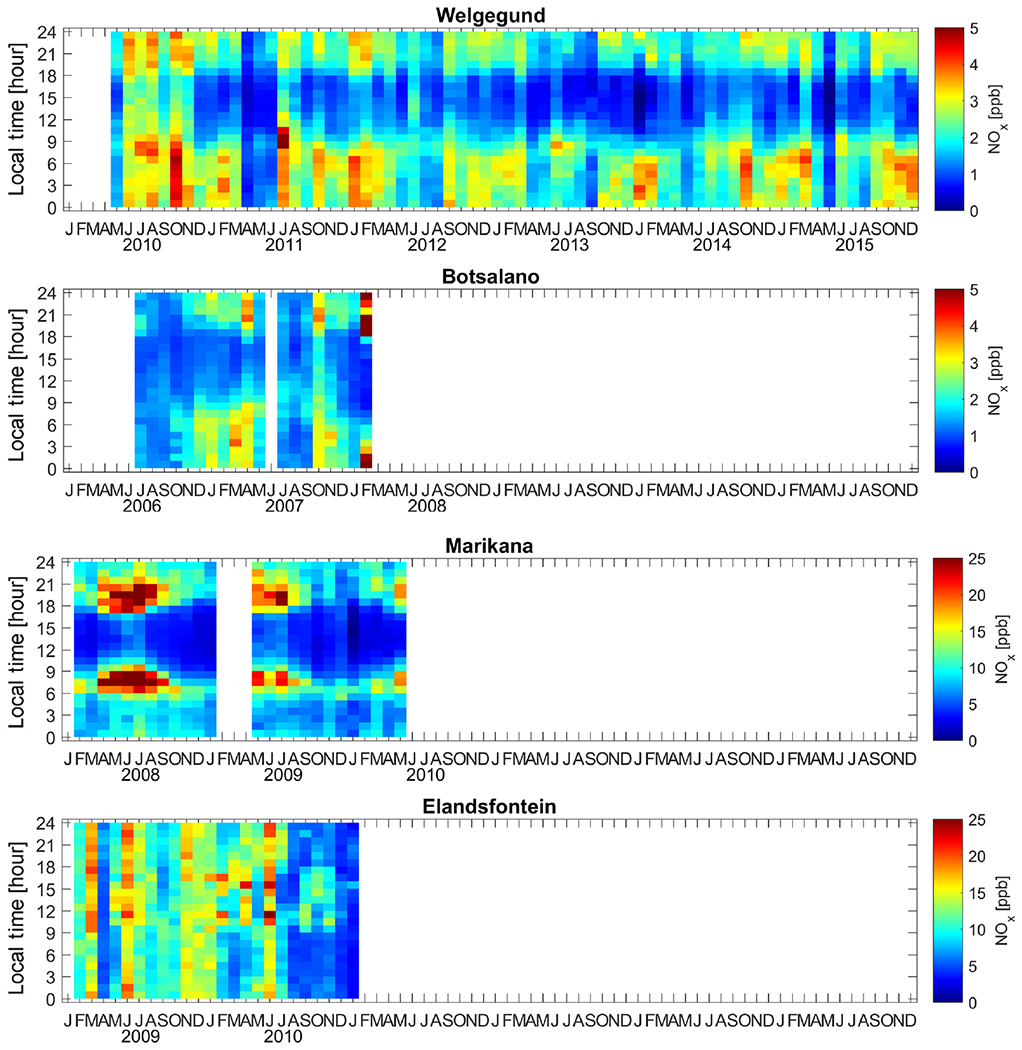
Time series of monthly median NO_x_ concentrations for each hour of the day at the four sites.

**Figure A8.**
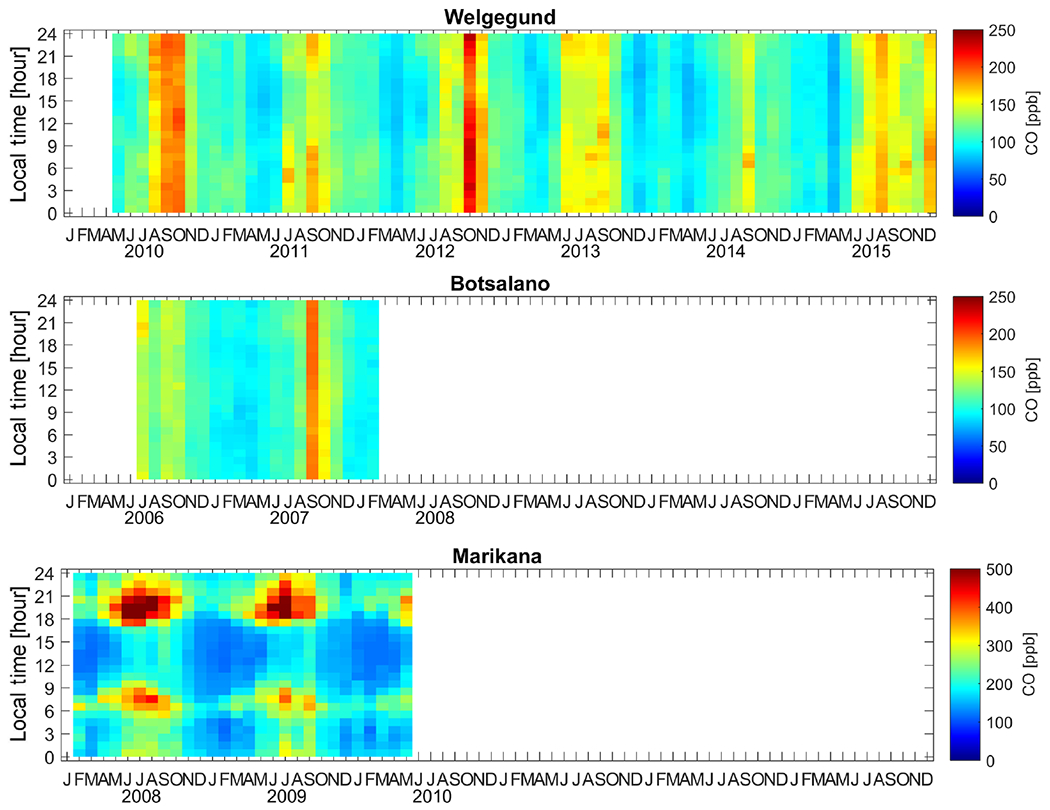
Time series of monthly median CO concentrations for each hour of the day at the four sites.

In Fig. 8, the correlations among O_3_, NO_*x*_, and CO concentrations at Welgegund, Botsalano, and Marikana are presented, which clearly indicate higher O_3_ concentrations associated with increased CO levels, while O_3_ levels remain relatively constant (or decrease) with increasing NO_*x*_. The highest O_3_ concentrations occur for NO_*x*_ levels below 10 ppb since the equilibrium between photochemical production of O_3_ and chemical removal of O_3_ shifts towards the former, i.e. greater O_3_ formation. In general, there seems to exist a marginal negative correlation between O_3_ and NO_*x*_ (Fig. A6) at all four sites, which is a reflection of the photochemical production of O_3_ from NO2 and the destruction of O_3_ through NO_*x*_ titration. These correlations among NO_*x*_, CO, and O_3_ indicate that O_3_ production in continental South Africa is limited by CO (and VOCs) concentrations, i.e. VOC limited.

This finding shows a strong correlation between O_3_ and CO and suggests that high O_3_ can be attributed to the oxidation of CO in the air masses; i.e. as long as there is a sufficient amount of NO_*x*_ present in a region, CO serves to produce O_3_. Although NO_*x*_ and VOCs are usually considered the main precursors in ground-level O_3_ formation, CO acts together with NO_*x*_ and VOCs in the presence of sunlight to drive photochemical O_3_ formation. According to Fig. 8, reducing CO emissions should result in a reduction in surface O_3_ and it is assumed that this response is analogous to that of VOCs. It is, however, not that simple since the ambient NO_*x*_ and VOC concentrations are directly related to the instantaneous rate of production of O_3_ and not necessarily to the ambient O_3_ concentration at a location, which is the result of chemistry, deposition, and transport that have occurred over several hours or a few days (Sillman, 1999). Notwithstanding the various factors contributing to increased surface O_3_ levels, the correlation between ambient CO and O_3_ is especially relevant given the low reactivity of CO with respect to ^•^OH radicals compared to most VOCs, which implies that the oxidation of CO probably takes place over a timescale of several days. It seems that the role of CO is of major importance in tropospheric chemistry in this region, where sufficient NO_*x*_ is present across continental South Africa and biogenic VOCs are relatively less abundant (Jaars et al., 2016), to fuel the O_3_ formation process.

#### 3.5.2 Seasonal change in O_3_–precursors relationship

**Figure 9.**
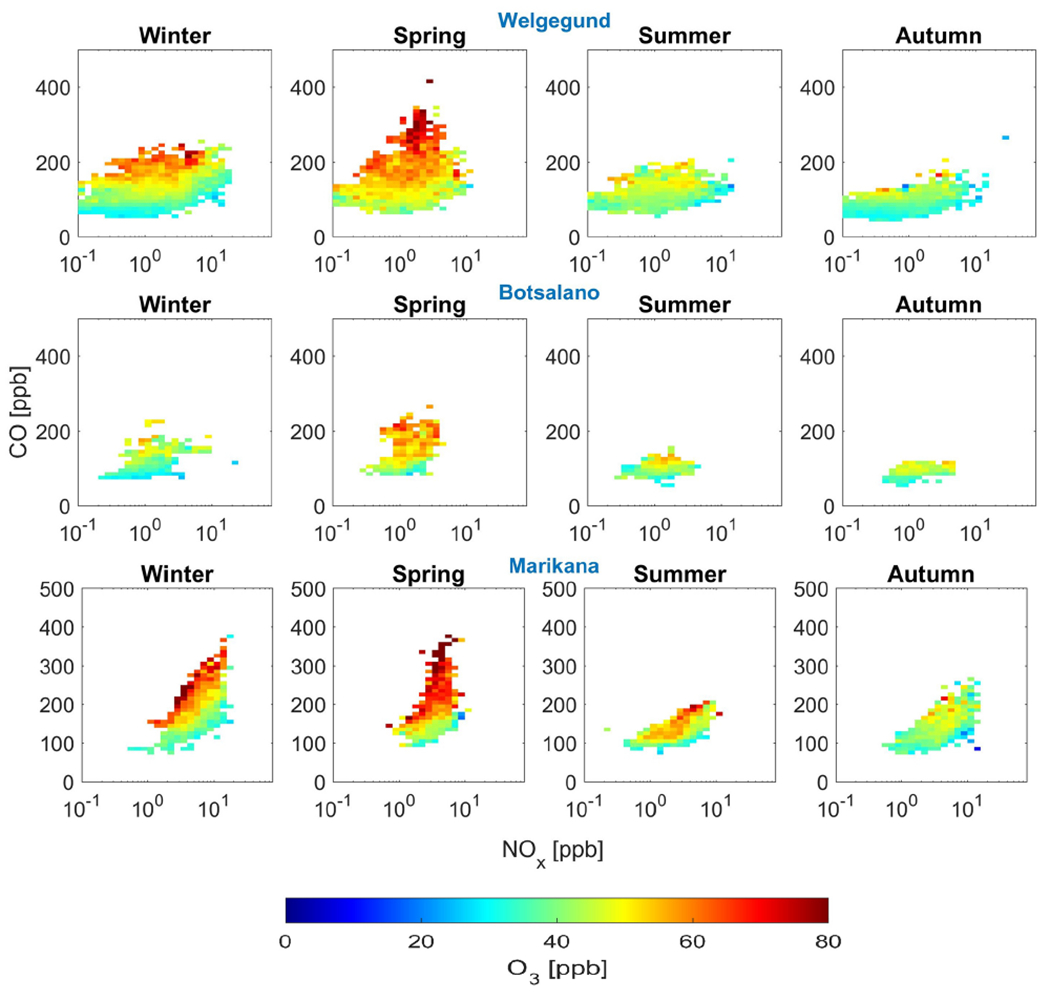
Seasonal plots of the relationship among O_3_, NO_*x*_, and CO at Welgegund, Botsalano, and Marikana.

Seasonal changes in the relationship between O_3_ and precursor species can be indicative of different sources of precursor species during different times of the year. In Fig. 9, the correlations between O_3_ levels and NO_*x*_ and CO are presented for the different seasons, which indicate seasonal changes in the dependence of elevated O_3_ concentrations on these precursors. The very high CO concentrations relative to NO_*x*_, i.e. high CO-to-NO_*x*_ ratios, are associated with the highest O_3_ concentrations, which are most pronounced (highest CO/NO_*x*_ ratios) during winter and spring. This indicates that the winter and spring O_3_ maximum is primarily driven by increased peroxy radical production from CO and VOCs. The seasonal maximum in O_3_ concentration coincides with the maximum CO concentration at the background sites, while the O_3_ peak occurs just after June–July when CO peaked at the polluted site Marikana (Fig. A5). This observed seasonality in O_3_ production signifies the importance of precursor species emissions from open biomass burning during winter and spring in this region, while household combustion for space heating and cooking is also an important source of O_3_ precursors, as previously discussed. The strong diurnal CO concentration patterns observed during winter at Marikana (Fig. A5) substantiate the influence of household combustion on CO levels, as indicated by Venter et al. (2012).

#### 3.5.3 O_3_ production rate

**Figure 10.**
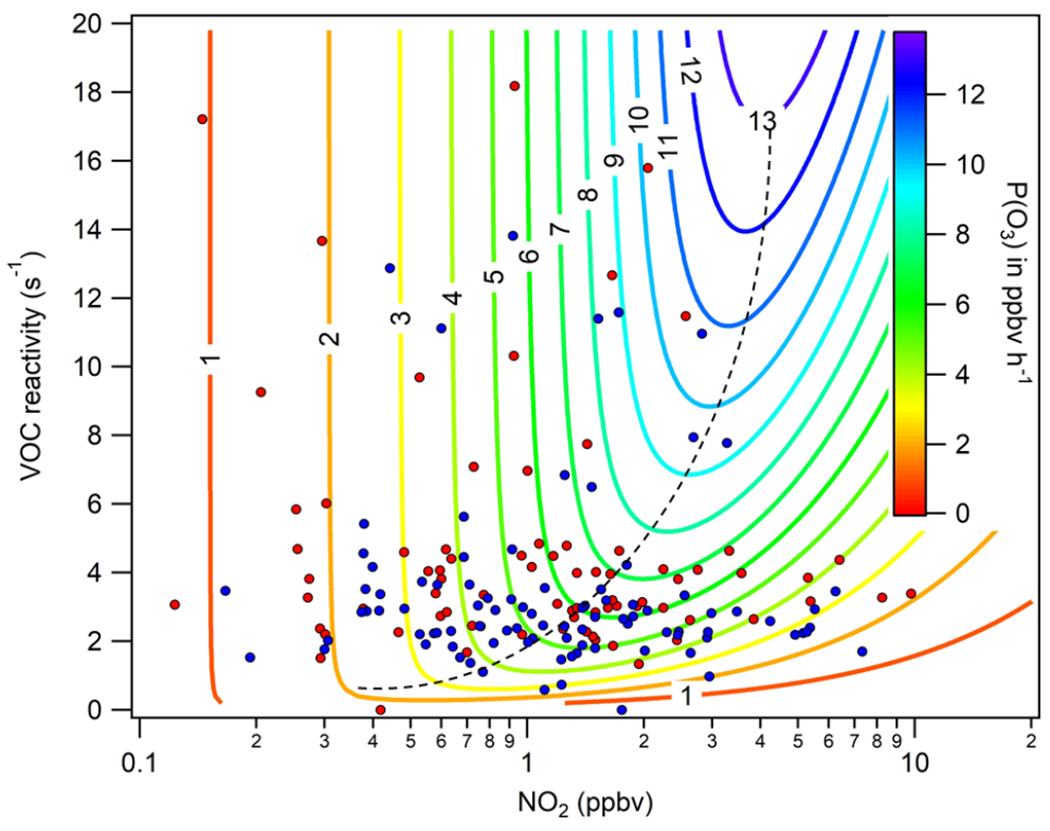
Contour plot of instantaneous O_3_ production (*P*(O_3_)) at Welgegund using daytime (11:00 LT) grab sample measurements of VOCs and NO_2_. The blue dots represent the first campaign (2011–2012), and the red dots indicate the second campaign (2014–2015).

In Fig. 10, *P*(O_3_) as a function of VOC reactivity calculated from the available VOC dataset for Welgegund (Sect. 2.4) and NO_2_ concentrations is presented. O_3_ production at Welgegund during two field campaigns, specifically at 11:00 LT, was found to range between 0 and 10 ppbv h^−1^. The average *P*(O_3_) values over the 2011 to 2012 and the 2014 to 2015 campaigns combined were 3.0±1.9 and 3.2±3.0 ppbv h^−1^, respectively. The dashed black line in Fig. 10, called the ridge line, separates the NO_*x*_- and VOC-limited regimes. To the left of the ridge line is the NO_*x*_-limited regime, when O_3_ production increases with increasing NO_*x*_ concentrations. The VOC-limited regime is to the right of the ridge line, when O_3_ production decreases with increasing NO_*x*_. According to the O_3_ production plot presented, approximately 40 % of the data are found in the VOC-limited regime area, which would support the regional O_3_ analysis conducted for continental South Africa in this study. However, the O_3_ production plot for Welgegund transitions between NO_*x*_ - and VOC-limited regimes, with Welgegund being in a NO_*x*_-limited production regime the majority of the time, especially when NO_*x*_ concentrations are very low (< 1 ppb). As indicated in Sect. 2.4, limitations to this analysis include limited VOC speciation data, as well as a single time-of-day grab sample. The O_3_ production rates can therefore only be inferred at 11:00 LT despite O_3_ concentrations peaking during the afternoon at Welgegund. Therefore, clean background air O_3_ production is most likely NO_*x*_ limited (Tiitta et al., 2014), while large parts of the regional background of continental South Africa can be considered VOC limited.

### 3.6 Implications for air quality management

#### 3.6.1 Ozone exceedances

**Figure 11.**
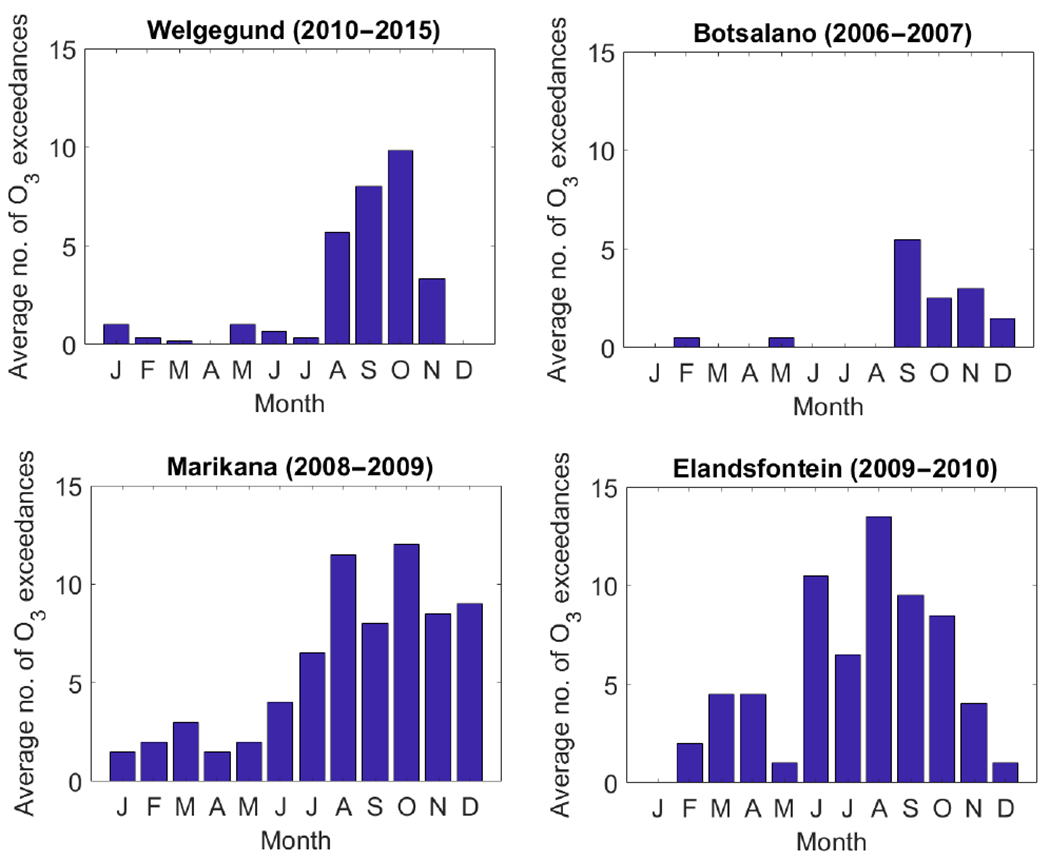
Monthly number of exceedances of the daily 8h O_3_ maximum (i.e. highest value of all available 8 h moving averages in that day) above 61 ppbv at Welgegund, Botsalano, Marikana, and Elandsfontein.

The South African National Ambient Air Quality Standard (NAAQS) for O_3_ is an 8 h moving-average limit of 61 ppbv with 11 exceedances allowed annually (Government Gazette, 2009). Figure 11 shows the average number of days per month when this O_3_ standard limit was exceeded at the four measurement sites. It is evident that the daily 8 h O_3_ maximum concentrations regularly exceeded the NAAQS threshold for O_3_ and the number of exceedances annually allowed at all the sites, including the most remote of the four sites, Botsalano. At the polluted locations of Marikana and Elandsfontein, the O_3_ exceedances peak early on in the dry season (June onwards), while at the background locations of Welgegund and Botsalano, the highest numbers of exceedances occur later in the dry season (August to November). These relatively high numbers of O_3_ exceedances at all the sites (background and industrial) highlight the regional O_3_ problem in South Africa, with background sites being impacted by the regional transport of O_3_ precursors from anthropogenic and biomass burning source regions.

#### 3.6.2 O_3_ control strategies

As indicated above (Sect. 3.4 and 3.5), O_3_ formation in the regions where Welgegund, Botsalano, and Marikana are located can be considered VOC limited, while the highly industrialized region with high NO_*x*_ emissions where Elandsfontein is located could also be considered VOC limited. Rural remote regions are generally considered to be NO_*x*_ limited due to the availability of NO_*x*_ and the impact of biogenic VOCs (BVOCs) (Sillman, 1999). However, Jaars et al. (2016) indicated that BVOC concentrations at a savannah grassland were at least an order of magnitude lower compared to other regions in the world. Therefore, very low BVOC concentrations, together with high anthropogenic emissions of NO_*x*_ in the interior of South Africa, result in VOC-limited conditions at background sites in continental South Africa.

It is evident that reducing CO and VOC concentrations associated with anthropogenic emissions, e.g. household combustion, vehicular emissions, and industries, would be the most efficient control strategy to reduce peak O_3_ concentrations in the interior of South Africa. It is also imperative to consider the seasonal variation in the CO and VOC source strength in managing O_3_ pollution in continental southern Africa. This study also revealed the significant contribution of biomass burning to O_3_ precursors in this region, which should also be considered when implementing O_3_ control strategies. However, since open biomass burning in southern Africa is of anthropogenic and natural origin, while O_3_ concentrations in continental South Africa are also influenced by trans-boundary transport of O_3_ precursors from open biomass burning occurring in other countries in southern Africa (as indicated above), it is more difficult to control. Nevertheless, open biomass burning caused by anthropogenic practices (e.g. crop residue, pasture maintenance fires, opening burning of garbage) can be addressed.

## 4 Conclusions

A spatial distribution map of O_3_ levels in the interior of South Africa indicated the regional O_3_ problem in continental South Africa, which was signified by the regular exceedance of the South African air quality standard limit. The seasonal and diurnal O_3_ patterns observed at the four sites in this study resembled typical trends for O_3_ in continental South Africa, with O_3_ concentrations peaking in late winter and early spring (see Zunckel et al., 2004), while daytime O_3_ corresponded to increased photochemical production. The seasonal O_3_ trends observed in continental southern Africa could mainly be attributed to the seasonal changes in emissions of O_3_ precursor species and local meteorological conditions. Increased O_3_ concentrations in winter at Welgegund, Marikana, and Elandsfontein reflected increased household combustion for space heating and the trapping of low-level pollutants near the surface. A spring maximum observed at all the sites was attributed to increased regional open biomass burning. Significantly higher O_3_ concentrations, which corresponded with increased CO concentrations, were associated with air masses passing over a region in southern Africa, where a large number of open biomass burning occurred from June to September. Therefore, the regional transport of CO associated with open biomass burning in southern Africa was considered a significant source of surface O_3_ in continental South Africa. A very small contribution from the stratospheric intrusion of O_3_-rich air to surface O_3_ levels at the four sites was indicated.

The relationship among O_3_, NO_*x*_, and CO at Welgegund, Botsalano, and Marikana indicated a strong correlation between O_3_ and CO, while O_3_ levels remained relatively constant (or decreased) with increasing NO_*x*_. Although NO_*x*_ and VOCs are usually considered to be the main precursors in ground-level O_3_ formation, CO can also drive photochemical O_3_ formation. The seasonal changes in the relationship between O_3_ and precursors species also reflected the higher CO emissions associated with increased household combustion in winter and open biomass burning in late winter and spring. The calculation of the *P*(O_3_) from a 2-year VOC dataset at Welgegund indicated that at least 40 % of O_3_ production occurred in the VOC-limited regime. These results indicated that large parts in continental South Africa can be considered VOC limited, which can be attributed to high anthropogenic emissions of NO_*x*_ in this region. It is, however, recommended that future studies should investigate more detailed relationships among NO_*x*_, CO, VOCs, and O_3_ through photochemical modelling analysis, while concurrent measurement of atmospheric VOCs and *OH would also contribute to the better understanding of surface O_3_ in this region.

In this paper, some new aspects of O_3_ for continental South Africa have been indicated, which must be taken into consideration when O_3_ mitigation strategies are deployed. Emissions of O_3_ precursor species associated with the concentrated location of industries in this area could be regulated, while CO and VOC emissions associated with household combustion and regional open biomass burning should also be targeted. However, emissions of O_3_ precursor species related to factors such as household combustion associated with poor socio-economic circumstances and long-range transport provide a bigger challenge for regulators.
